# Breakthrough viridans streptococcal bacteremia in allogeneic hematopoietic stem cell transplant recipients receiving levofloxacin prophylaxis in a Japanese hospital

**DOI:** 10.1186/s12879-016-1692-y

**Published:** 2016-08-05

**Authors:** Muneyoshi Kimura, Hideki Araoka, Atsushi Yoshida, Hisashi Yamamoto, Masahiro Abe, Yuki Okamoto, Mitsuhiro Yuasa, Daisuke Kaji, Kosei Kageyama, Aya Nishida, Kazuya Ishiwata, Shinsuke Takagi, Go Yamamoto, Yuki Asano-Mori, Naoyuki Uchida, Akira Hishinuma, Koji Izutsu, Atsushi Wake, Shuichi Taniguchi, Akiko Yoneyama

**Affiliations:** 1 Department of Infectious Diseases, Toranomon Hospital, 2-2-2 Toranomon, Minato-ku, Tokyo, 105-8470 Japan; 2 Department of Infection Control and Clinical Laboratory Medicine, Dokkyo Medical University, Tochigi, Japan; 3 Department of Hematology, Toranomon Hospital, Tokyo, Japan; 4 Okinaka Memorial Institute for Medical Research, Tokyo, Japan

**Keywords:** Allogeneic hematopoietic stem cell transplantation, Febrile neutropenia, Levofloxacin prophylaxis, Levofloxacin breakthrough, Ceftazidime, Viridans streptococcus

## Abstract

**Background:**

Breakthrough viridans streptococcal bacteremia (VSB) in patients with hematological malignancy receiving levofloxacin prophylaxis is a major blood stream infection (BSI) occurring during febrile neutropenia. However, clinical data focused on VSB in allogeneic hematopoietic stem cell transplant (allo-HSCT) recipients are lacking.

**Methods:**

The medical records of allo-HSCT recipients who received oral levofloxacin prophylaxis between January 2011 and August 2013 at Toranomon Hospital were reviewed to evaluate breakthrough VSB. Stored viridans streptococcal (VGS) species were identified by using sod*A* gene sequencing, and were assessed for drug susceptibility.

**Results:**

Among the 184 allo-HSCT recipients on levofloxacin prophylaxis, 28 (15.2 %) experienced breakthrough VSB. All of the 28 recipients with VSB were treated with a cefepime-based or piperacillin/tazobactam-based regimen. The susceptibility rates of the VGS strains for levofloxacin, cefepime, piperacillin/tazobactam, meropenem, and vancomycin were 0 %, 95 %, 100 %, 100 %, and 100 %, respectively. Both the MIC_50_ (minimum inhibitory concentration) and the MIC_90_ of ceftazidim (0.5 μg/mL and 2 μg/mL, respectively) were higher than the MIC_90_ of all the other anti-pseudomonal beta-lactams (APBLs). Only 1 VGS strain had a penicillin MIC ≥ 2 μg/mL by the Etest (3.6 %). There were no cases with acute respiratory distress syndrome (ARDS) that was associated with VSB, although the rate of viridans group streptococcal shock syndrome was high (26 %). The crude 30-day mortality rate in the VSB group (10.7 %) did not differ significantly from that in the BSI without VSB group (9.3 %) or non-BSI group (7.0 %) (*P* = 0.77). Also, VSB was not a risk factor for all-cause mortality up to 60 days following allo-HSCT (*P* = 0.43).

**Conclusions:**

APBL with increased anti-VGS activity (APBL-VA) monotherapy would typically be optimal for treating the VGS strains in this setting. Indication of adding an empiric anti-gram-positive agent to APBL-VA for treating VSB should depend on local factors, such as the susceptibility results. In addition, breakthrough VSB is probably not a major cause of death in allo-HSCT settings, where beta-lactam non-susceptible VGS and the ARDS are rare.

**Electronic supplementary material:**

The online version of this article (doi:10.1186/s12879-016-1692-y) contains supplementary material, which is available to authorized users.

## Background

Fluoroquinolone prophylaxis should be considered for high-risk patients with prolonged and profound neutropenia [1]. Allogeneic hematopoietic stem cell transplant (allo-HSCT) recipients are the primary constitutes of this high-risk group. Among them, breakthrough blood stream infection (BSI) on fluoroquinolone prophylaxis has been reported [2–5]. Some allo-HSCT recipients have also been reported to have breakthrough BSI on levofloxacin prophylaxis, and viridans group streptococcus (VGS) is one of the causative organisms [2, 3]. Additionally, VGS is a major causative organism of febrile neutropenia (FN) in cancer patients [6–10]. The 2010 Infectious Diseases Society of America (IDSA) FN guideline recognizes that anti-pseudomonal beta-lactam (APBL) antibiotics currently employed as empiric monotherapy in FN generally have good in vitro anti-VGS activity [1]. The APBLs with increased anti-VGS activity (APBL-VAs) recommended by the guideline are cefepime, piperacillin/tazobactam, and antipseudomonal carbapenems [1], and as such anti-gram-positive agents (AGPAs), including vancomycin, linezolid, or daptomycin, are not routinely recommended when viridans streptococcal bacteraemia (VSB) is suspected [1]. However, it is not clear whether this recommendation is optimal for all clinical settings, including the allo-HSCT setting. A recent report described that in vitro resistance to the APBL-VAs was observed in VGS isolates with a penicillin minimum inhibitory concentration (MIC) measured by Etest of ≥ 2 μg/mL [11]. Therefore, the clinical criteria that can assist with the targeted use of AGPAs only for the VSB that caused by VGS strains with penicillin MIC ≥ 2 μg/mL has been suggested [11]. On the other hand, some clinical centers choose prophylactic vancomycin for high-risk patients to prevent infection caused by gram-positive cocci such as VGS [12, 13]. A report from United States described that the mortality rate of VSB was 21.9 % and early administration of vancomycin was highly protective against VSB in the allo-HSCT setting [13]. However, the allo-HSCT recipients did not receive any prophylactic antimicrobial agents such as fluoroquinolone, and were initially treated by combination of ticarcillin/clavulanic acid and amikacin during neutropenia [13]. These findings may not extend to current allo-HSCT settings.

This is the first report to describe the characteristics of VSB and therapeutic strategies for the VSB in allo-HSCT recipients with FN who received levofloxacin prophylaxis in the era of APBL-VAs.

## Methods

### Study patients

A retrospective analysis of VSBs among allo-HSCT recipients (age, ≥16 years) who received standard prophylaxis with 500 mg/day oral levofloxacin was conducted between January 2011 and August 2013 at the Toranomon Hospital (890 beds; Tokyo, Japan). The medical and microbiological records of the recipients under levofloxacin prophylaxis during the study period were reviewed. Our standard strategy of levofloxacin prophylaxis for allo-HSCT recipients was introduced in January 2011 comprises following three major policies: (1) 500 mg/day oral levofloxacin prophylaxis started before day −7 (7 days before allo-HSCT); (2) levofloxacin prophylaxis stopped at the first episode of FN and changed to another antimicrobial regimen immediately after obtaining ≥ 2 sets of blood cultures; and (3) continuation of levofloxacin prophylaxis until neutrophil engraftment in the absence of any FN episodes. Neutrophil engraftment was defined as the first of 3 consecutive days with ANC (absolute neutrophil count) of ≥ 500cells/μL. A central venous access device was inserted before conditioning was started. All recipients received antifungal agents and acyclovir for prophylaxis. Trimethoprim-sulfamethoxazole was given from day −7 through day −2 for *Pneumocystis* prophylaxis. Urine, stool, nasal, and pharyngeal swabs were collected from each allo-HSCT recipient for methicillin-resistant *Staphylococcus aureus* (MRSA) colonization screening within 3 months before allo-HSCT. Stool samples were also collected for vancomycin-resistant enterococci (VRE) colonization screening at the same time. All the cord blood transplant recipients received single cord blood during the study period.

This study was approved by the Human Ethics Review Committee of Toranomon Hospital.

### Definitions

Breakthrough VSB was defined as the presence of at least a single positive result of blood culture for VGS in a sample drawn from a patient at the first episode of FN under levofloxacin prophylaxis. The definition of VSB was the same as the previous study in the allo-HSCT setting [13].

The definition of FN included criteria for body temperature and neutrophil count: Body temperature ≥ 37.5 °C measured at the axially fossa, according to routine practice in Japan [14]; and ANC <500 cells/μL or ANC that decreased to <500 cells/μL during the 48 h following the occurrence of fever [1].

For disease status, all hematological disorders were defined as either standard risk, or high risk [15]. Conditioning regimens were classified based on the report by the Center for International Blood and Marrow Transplant Research [16]. Myeloid malignancy contains acute myeloid leukemia (AML), myelodysplastic syndrome (MDS), MDS overt AML, and chronic myelogenous leukemia. The recipients with prior history of allogeneic hematopoietic stem cell transplantation (PH-allo-HSCT) indicates the recipients who received allogeneic hematopoietic stem cell transplantation 2 or more times. Coagulase negative staphylococci, Corynebacterium species, unidentified gram positive cocci, and unidentified gram positive cocci were considered contaminants unless they were cultured from ≥ 2 separate blood culture bottles. Co-infection was defined as the identification of ≥ 2 bacterial species in multiple blood culture bottles of samples collected within 24 h.

Hypotension, septic shock, and acute respiratory distress syndrome (ARDS) were defined as described previously [17, 18]. Refractory hypotension was defined as hypotension that was refractory to intravenous fluid therapy. VGS shock syndrome was defined as monobacteremia of viridans streptococcus (mono VSB) with refractory hypotension (patients who had VSB with co-infection were excluded). Invasive aspergillosis was defined as probable or proven invasive aspergillosis according to the EORTC-MSG criteria [19].

### Initial antimicrobial regimen at the onset of levofloxacin breakthrough FN

Monotherapy with APBL-VA, as recommended by the IDSA guideline, is the routine empiric initial antimicrobial regimen administered at the onset of FN [1]. Clinicians could choose any of the APBL-VAs including cefepime, piperacillin/tazobactam, and meropenem. In our institution, AGPAs and amikacin were not routinely administered as a standard component of the empiric initial antimicrobial regimen for FN.

AGPAs should be combined with the APBL-VA regimen for specific clinical indications, including suspected catheter-related infection, skin or soft-tissue infection, or severe sepsis which caused by beta-lactam-resistant gram-positive organisms. Clinicians usually chose vancomycin as the first line AGPAs, except when the recipients were allergic to vancomycin. Amikacin could be combined with an APBL-VA regimen for suspected severe sepsis caused by beta-lactam-resistant gram-negative rod.

### Identification of VGS and other organisms

Strains of VGS were initially identified from colony morphology and by hemolysis and the VITEK2 system (bioMérieux, Marcy l’Etoile, France). Additionally, the stored VGS strains obtained from the blood samples between August 2011 and August 2013 were also identified to species level by examining the heterogeneity in streptococcal *sod A* gene sequences [20]. General identification of the other organisms that cultured from blood samples to species level was performed by VITEK2 (bioMérieux, Marcy l’Etoile, France), or WalkAway 96 SI (Siemens Healthcare, Deerfield, IL, USA). *Helicobacter cinaedi* was identified by using gyrB-targeted PCR methods. *Corynebacterium* spp. was identified into species level by RapID CB Plus system (Remel, Lenexa, KS, USA).

### Antimicrobial susceptibility

Drug susceptibility tests for levofloxacin, ceftazidime, cefepime, piperacillin/tazobactam, meropenem, and vancomycin were performed using microdilution methods by the Clinical Laboratory and Standards Institute (CLSI) at Dokkyo Medical University for the stored VGS strains obtained from the blood samples between August 2011 and August 2013 [21]. In addition, tests for penicillin were performed using Etest (bioMérieux, Durham, NC, USA) for all the VGS strains obtained at Toranomon Hospital during the entire study period. Drug susceptibility breakpoints in VGS species, as established by CLSI M100-S22, were used to categorize the MICs of the cultured VGS strains [21]. Susceptibility guidelines for VGS are not available for piperacillin/tazobactam; therefore, a cutoff MIC of 16/4 μg/mL was chosen on a previous report [11]. The breakpoint of ceftazidime has not been defined in the CLSI M100-S22 [21].

### Outcome evaluation of the febrile neutropenia

An evaluation cohort (EC) was created to evaluate the clinical characteristics of breakthrough VSB as compared with other groups at the first episode of FN. Among all the study subjects, the allo-HSCT recipients who experienced FN were included into the EC; the recipients who successfully achieved neutrophil engraftment without FN and continued levofloxacin prophylaxis until neutrophil engraftment were excluded from the EC. The EC subjects were then divided into three groups: VSB group, consisting of recipients with VSB; BSI without VSB group, comprising the recipients with BSI where the causative organisms was not VGS; and the non-BSI group, comprising recipients without BSI at the first episode of FN. Recipients with contaminant organisms cultured from the blood samples included into non BSI group.

### Statistical analysis

Categorical variables were compared by the Fisher’s exact test. Continuous variables from the different three groups were compared by Kruskal-Wallis test. The 30-day mortality rates and the 60-day mortality rates after the onset of FN were estimated using Kaplan-Meier analysis and the groups were compared using log-rank test. The incidence of VSB was estimated based on cumulative incidence curves. Competing event was febrile neutropenia that associated with non VSB causes. The groups were compared using Gray’s test. For the multivariate analysis, variables that *P*-values showed ≤ 0.10 were entered into the Fine-Gray proportional hazard model and sequentially eliminated in a stepwise backward fashion until the remaining variables were statistically significant. Log-rank test and proportional hazard model were used for pre-transplant variables and time dependent variables (VSB and engraftment within 30 days following allo-HSCT), respectively to identify the risk factors associated with 60 days overall mortality following allo-HSCT in the univariate analysis. For the multivariate analysis, valuables that *P*-values showed ≤ 0.10 were entered into the proportional hazard model and sequentially eliminated in a stepwise backward fashion until the remaining factors were statistically significant. Significance was set at α = .05. All statistical analysis was performed with EZR (Saitama Medical Center, Jichi Medical University), which is a graphical user interface for R (The R Foundation for Statistical Computing) [22].

## Results

### Patient characteristics

Among the 184 allo-HSCT recipients who received the standard levofloxacin prophylaxis during the study period, 28 had breakthrough VSB. Twenty four of the 28 VGS strains (86 %) were cultured from ≥2 sets of blood culture bottles. The cumulative incidence of the VSB was 15.2 %. The characteristics of the 28 recipients are shown in Table 1. Among them, 22 received cord blood stem cell transplantation, 4 received related peripheral blood stem cell transplantation, and 2 received unrelated bone marrow transplantation.

**Table 1:** Clinical and microbiological characteristics of 28 breakthrough VSB cases

Characteristics	No.
Median age (range)	55 (20–71)
Gender	
Male	15 (53.6 %)
Female	13 (46.4 %)
Hematological disorder	
Myeloid malignancy	
AML	3 (10.7 %)
MDS overt AML	6 (21.4 %)
MDS	8 (28.6 %)
CML	1 (3.6 %)
Lymphoid malignancy	
ALL	5 (17.9 %)
NHL	1 (3.6 %)
T-PLL	1 (3.6 %)
Others	
Others	3 (10.7 %)
Hematopoietic stem cell transplantation	
CBT	22 (78.6 %)
rPBSCT	4 (14.3 %)
uBMT	2 (7.1 %)
Status of the patients	
Nosocomial infection	28 (100 %)
Severe neutropenia (ANCs <100/pL )	28 (100 %)
Receipt of beta-lactam in the previous 30 day	6 (21.4 %)
Clinical presentation	
Median body temperature (range) (°C)	38.6 (37.5-40.6)
Acute respiratory distress syndrome	0
Co-infection	9 (32.1 %)
Co-infectious pathogen	
Staphylococcus epidermidis	5
Corynebacterium striatum	2
Enterococcus faecium	1
Rothia mucilaginosa	1
Escherichia coli	1
Treatment	
Cefepime	26 (92.9 %)
Piperacillin/tazobactam	2 (7.14 %)
Empiric VCM administration	4 (14.3 %)
Clinical outcome	
Crude 30-day mortality	3 (10.7 %)
Crude 60-day mortality	3 (10.7 %)
Clinical presentation of mono VSB	*N* = 19
VGS shock syndrome	5 (26 %)

*AML* acute myeloid leukemia, *MDS* myelodysplastic syndrome, *CML* chronic myelogenous leukemia, *ALL* acute lymphoblastic leukemia, *NHL* non Hodgkin lymphoma, *T-PLL* T-cell prolymphocytic leukemia, *SAA* severe aplastic anemia, *CBT* cord blood transplantation , *rPBSCT* related peripheral blood cell transplantation, *uBMT* unrelated bone marrow transplantation, *ANCs* absolute neutrophil counts, *VCM* vancomycin, *VSB* viridans streptococcal bacteremia, *VGS* viridans group streptococcus

### Microbiological characteristics of the breakthrough VSB

The clinical and microbiological characteristics of breakthrough VSB are shown in Table 1. All 28 VSBs occurred as nosocomial infections during severe neutropenia (ANCs ≤ 100/μL) before neutrophil engraftment (from 2 days before allo-HSCT to 8 days after allo-HSCT). Nine recipients had co-infections (32 %) caused by 10 organisms, which are listed in Table 1. Overall, 22 of the 28 VGS strains were obtained between August 2011 and August 2013 and stored; the remaining 6 VGS strains were obtained from January 2011 to July 2013 but not stored. Among the 22 stored VGS strains, the causative strains identified by *sodA* signature sequences were *S. mitis* (14 strains), *S. oralis* (4 strains), *S. australis* (1 strain), and *S. infantis* (1 strain); 2 strains could not be identified. Of these, only *S. mitis* is capable of causing VGS shock syndrome. Then, 5 of the 14 *S. mitis* (36 %) caused VGS shock syndrome.

The results of antimicrobial susceptibility tests for beta-lactam agents are shown in Table 2. Among the 22 stored VGS strains, the susceptibility rates for levofloxacin, cefepime, piperacillin/tazobactam, meropenem, and vancomycin were 0 %, 95 %, 100 %, 100 %, and 100 %, respectively. Both the MIC_50_ of ceftazidime and MIC_90_ of ceftazidime were higher than those of the APBL-VAs. Additionally, only 1 of the 28 VGS strains (3.6 %) had a penicillin MIC 2 μg/mL by Etest. The penicillin MICs for the remaining VGS strains were <2 μg/mL. The penicillin MICs for all the 6 VGS strains that cultured from blood samples obtained from the recipients who were exposed to a beta lactam in the previous 30 days (Table 1) were ranged from 0.03 to 0.25 μg/mL (<2 μg/mL) by Etest.

**Table 2:** Antimicrobial (beta lactam) susceptibility of the breakthrough viridans group streptococcal strains

								No. of isolates at each MIC, mg/L									
Antimicrobial agent	No. of isolates tested	Susceptible range mg/L	No. of the susceptible strains	Resistant range mg/L	No. of the resistant strains	MIC_50_, mg/L	MIC_90_, mg/L	≤0.03	0.06 ≤0.06; CFPM	0.12	0.25 ≤0.25; CAZ	0.5	1	2	4	8	16
Penicillin	28	≤0.12	22 (79 %)	>4	0	0.06	0.25	8	9	5	3	2	0	1	0	0	0
Ceftazidime	22	NA	NA	NA	NA	0.5	2				5	7	7	1	0	2	0
Cefepime	22	≤1	21 (95 %)	>4	0	0.12	0.25		9	7	4	0	1	1	0	0	0
Meropenem	22	≤0.5	22 (100 %)	>1	0	≤0.03	0.12	17	2	2	1	0	0				
											≤0.25/4	0.5/4	1/4	2/4	4/4	8/4	>16/4
Piperacillin/tazobactam	22	≤8/4	22 (100 %)	>16/4	0	≤0.25/4	≤0.25/4				21	1	0	0	0	0	0

*MIC* minimum inhibitory concentration, *CFPM* cefepime, *CAZ* ceftazidim, *NA* not available

### Risk factors of the breakthrough VSB

To identify risk factors, the cumulative incidences of the VSB among each group were compared (Additional file.1). The recipients with the high risk hematological disorders tended to have the VSB more frequent than those with the standard risk (*P* = 0.11). However, no significant risk factors were identified by univariate analysis and multivariate analysis.

### Therapeutic regimen

For empirical therapy, all 28 recipients received a cefepime-based regimen (26 recipients) or a piperacillin/tazobactam-based regimen (2 recipients) immediately after withdrawing levofloxacin prophylaxis at the first episode of FN. Four recipients received empirical vancomycin administration. None of the recipients received any empirical AGPA other than vancomycin.

### Outcome of the breakthrough VSB in the evaluation cohort

Among 19 recipients with mono VSB, 5 had VGS shock syndrome (26 %). However, none of the recipients experienced ARDS. The crude 30-day mortality rate of the VSB was 10.7 %. All 6 recipients who had septic shock survived. In the 3 recipients who died, all the causative VGS strains were susceptible to penicillin (MIC ≤ 0.12 μg/mL).

**Fig. 1:**
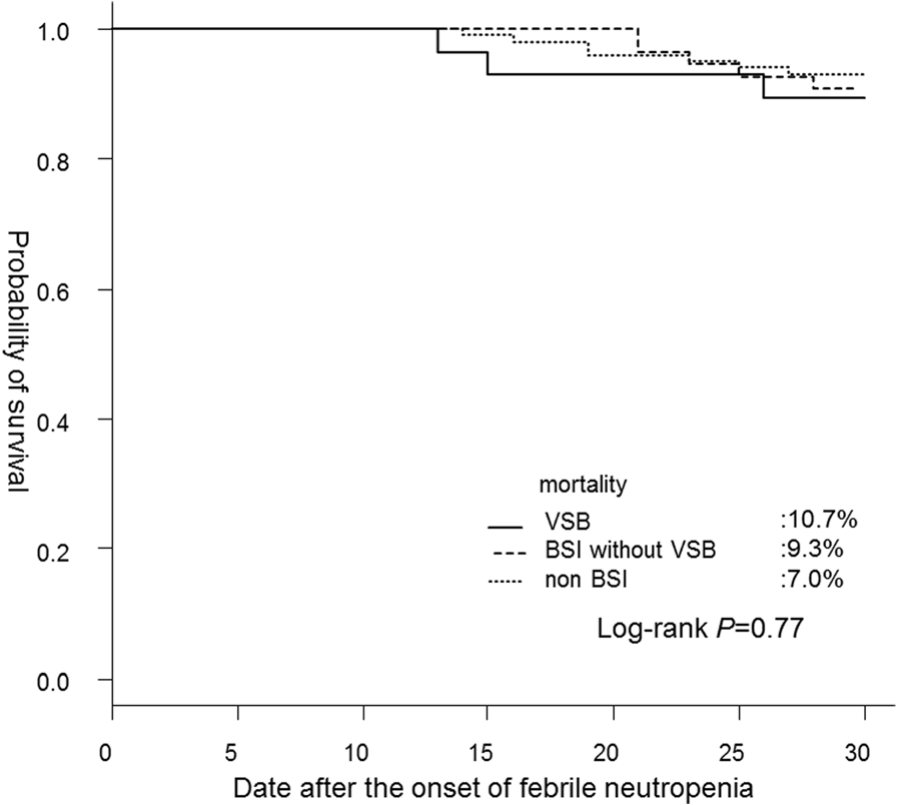
The crude 30-day mortality after the onset of febrile neutropenia among the three groups

Next, the characteristics and outcome of VSB in the EC were evaluated. The EC consisted of 182 recipients, since 2 recipients were excluded because they successfully achieved neutrophil engraftment without FN and continued levofloxacin prophylaxis until neutrophil engraftment. All the recipients were started on an APBL-VA-based regimen at the onset of FN instead of levofloxacin prophylaxis (Additional file.2). Thirteen recipients (7.1 %) received additional empiric vancomycin administration along with the APBL-based regimen. All the recipients in the EC were divided into the VSB group (28 patients), BSI without VSB group (54 patients), or non-BSI group (100 patients). Some minor differences were observed among the groups among the three groups (Additional file.2). The causative organisms in the BSI without VSB group are shown in Table 3. Among the 54 recipients in the BSI without VSB group, 5 had co-infections. At the onset of FN, 1 recipient had MRSA bacteremia and 1 had micafungin breakthrough fungemia caused by *Trichosporon asahii*; however, none of the recipients had VRE bacteremia or invasive aspergillosis. Clinicians ordered colonization screening tests for 165 of the 182 recipients in the EC (91 %). The rate of MRSA colonization and VRE colonization before transplantation were 3 % (5/165), and 0 % (0/165), respectively. The crude 30-day mortality rate after the onset of FN in the VSB group (10.7 %) was not significantly different from that in the BSI without VSB group (9.3 %) or the non-BSI group (7.0 %) (*P* = 0.77) (Fig. 1). Also, the crude 60-day mortality rate in the VSB group (10.7 %) was not significantly different from that in the BSI without VSB group (16.7 %) or the non-BSI group (14.0 %) (*P* = 0.81).

**Table 3:** Causative organisms in the BSI without VSB group as determined from blood cultures

	No of strains
Gram positive cocci	
Coagulase negative staphylococcus	19
*Enterococcus faecium*	4
*Enterococcus faecalis*	2
MRSA	1
*Streptococcus agalactiae*	1
Unidentified GPC	2
Gram positive rods	
*Corynebacterium striatum*	10
*Corynebacterium jeikeium*	1
*Corynebacterium* spp.	1
Unidentified GPR	2
Gram negative rods	
*Escherichia coli*	10
*Pseudomonas aeruginosa*	2
*Klebsiella pneumoniae*	1
*Campylobacter* spp.	1
*Helicobacter cinaedi*	1
Fungi	
*Trichosporon asahii*	1

All the gram negative rods were resistant to levofloxacin except for *Helicobacter cinaedi* and *Campylobacter* spp. The standard drug susceptibility tests for *Helicobacter cinaedi* and Campylobacter spp. could not be performed in our institute

*BSI* blood stream infection, *VSB* viridans streptococcal bacteremia, *GPC* gram-positive coccus, *GPR* gram-positive rod

### Risk factors for all-cause mortality up to 60 days following allo-HSCT

Engraftment within 30 days following allo-HSCT, high risk hematological diseases, and PH-allo-HSCT were associated with the outcome up to 60 days following allo-HSCT significantly in univariate analysis (Table 4). While, VSB was not identified as the risk factor for the mortality (*P* = 0.43). Then, engraftment within 30 days was the independent factor that improved the survival significantly (*P* = 0.0014) in the multivariate analysis (Table 4).

**Table 4:** Risk factor for all-cause mortality up to 60 days following allogeneic hematopoietic stem cell transplantation

Univariate analysis			
Pre-transplant factors	Variables (Number)	Mortality (%)	*P*-value
Gender	Male (119)	19.3	0.13
	Female (65)	10.8	
Age	≥60 (80)	18.8	0.42
	<60 (104)	14.4	
Diagnosis	Myeloid malignacy (119)	16.8	0.83
	non Myeloid malignancy (65)	15.4	
Diseases risk	High risk hematological diseases (151)	19.2	0.028
	Standard risk hematological diseases (33)	3.03	
PH of allo-HSCT	Yes (25)	32.0	0.017
	No (159)	13.8	
Donor typre	CBT (135)	19.3	0.076
	non CBT (49)	8.16	
Conditioning	RIC(63)	22.3	0.11
	MAC (121)	13.2	
Time dependent factors			
Viridans streptococcal bacteremia	VSB (28)	10.7	0.43
	non VSB (156)	17.3	
Engraftment within Day30	Yes (161)	9.94	<0.001
	No (23)	60.9	
Multivariate analysis			
Factors	Hazard ratio	95 % confidence interval	*P*-value
High risk hematological diseases	5.59	0.741–42.18	0.095
Prior history of allo-HSCT	2.09	0.919–4.740	0.079
CBT	1.66	0.569–4.839	0.35
Engraftment within Day30	0.22	0.087–0.558	0.0014

*CBT* cord blood transplantation, *RIC* reduced-intensity conditioning, *MAC* Myeloablative conditioning, *allo-HSCT* allogeneic hematopoietic stem cell transplantation, *VSB* viridans streptococcal bacteremia, *PH-allo-HSCT* prior history of allogeneic hematopoietic stem cell transplantation

## Discussion

The high incidence of levofloxacin breakthrough VSB in this study (15.2 %) was comparable to that of the previous study conducted in autologous peripheral blood stem cell transplant setting (16.2 %) [6]. However, it was higher than those in other studies that described levofloxacin breakthrough BSI in allo-HSCT setting [2, 3]. Fluoroquinolone prophylaxis had been approved for approximately 10 years at our hospital (Tosufloxacin prophylaxis was used in our institute before January 2011 when the levofloxacin prophylaxis was started instead of tosufloxacin prophylaxis.). The long use of the fluoroquinolone prophylaxis might select VGS with diminished susceptibility to levofloxacin [6].

None of the 28 VGS strains assessed in this study were susceptible to levofloxacin; in contrast, none of the strains were resistant to penicillin based on the breakpoint of CLSI (penicillin MIC ≥ 4 μg/mL) [21]. This low penicillin resistant rate (0 %) was comparable to that reported in Finland (2.3 %) [23]. In addition, the penicillin MIC was 2 μg/mL for only 1 VGS strain, as assessed by Etest (Table 2). Therefore, based on a previous report, only 1 of the 28 VGS strains (3.6 %) was not considered to have good susceptibility to APBL-VAs [11]. This rate of 3.6 % for a penicillin MIC ≥ 2 μg/mL differs considerably from the rates observed in North America and Spain, which ranged from 17 % to 22 % [11, 24, 25]. Moreover, the susceptible rates of the APBL-VAs in this study were over 95 %. In contrast, in vitro inferiority of ceftazidime compared with the APBL-VAs was shown (Table 2). A similar result was reported in the previous study [24]. Accordingly, ceftazidime should probably not be administrated as a first line empirical therapeutic agent instead of the APBL-VAs when clinicians treat levofloxacin breakthrough FN in a setting, where breakthrough VSB is prevalent.

In the present study, 21 of the 24 recipients who did not receive empiric vancomycin administration at the first episode of FN were added vancomycin to APBL-VA-based regimen immediately after a gram-positive coccus was cultured from blood samples. This practice is recommended by the IDSA guideline [1]. However, according to the in vitro data described above, APBL-VA mono therapy was optimal for almost all the VGSs identified in our study. Thus, the empiric addition of vancomycin to APBL-VA-based regimen would not typically be needed for treating mono VSB in our setting, even when clinicians are awaiting the drug susceptibility results of VGS strains.

Nosocomial VSB and receipt of a beta lactam antimicrobial in the previous 30 days were reported to be the useful factors to predict VGS strains with a penicillin MIC ≥ 2 μg/mL in the previous study that conducted in the setting where beta-lactam non-susceptible VGS strains were prevalent [11]. However, the positive predicted values of them in the present study were only 3.7 % (1/28), and 0 % (0/6), respectively. Hence, the predictive factors in the previous study would probably lead to overuse of unnecessary empiric AGPAs administration in the settings where beta-lactam non-susceptible VGS strains were rare.

In this study, both the crude 30-day mortality rate and the crude 60-day mortality was 10.7 %. These were lower than those reported in previous studies in the allo-HSCT settings, which ranged from 21.9 to 24 % [13, 26]. In the previous study, seven of the 32 recipients with VSB (21.9 %) died, at median of 26 days (range, 12–36 days) from VSB diagnosed because of ARDS and multiorgan failure [13].

Further, both the crude 30-day mortality and the crude 60-day mortality after the onset of FN did not differ significantly from those in the other two groups in this study. Also, VSB was not a risk factor for all-cause mortality up to 60 days following allo-HSCT (Table 4). There was no VSB with ARDS in the present study, although 3 % to 33 % of VSB caused ARDS during neutropenia in the previous studies [7]. It was probably one of the reasons why the mortality of VSB in our study was lower. *S. mitis* cluster2 identified by multilocus sequence analysis is probably associated with ARDS because it was reported to be associated with unexplained pulmonary infiltrate during neutropenia [27]. There might be few *S. mitis* cluster2 among all the 28 VGS strains in our study. Additionally, the previous studies were not conducted in the era of APBL-VAs, which may be one of the reasons underlying the high mortality. However, the exact reasons for the difference are unclear since the drug susceptibility data from these studies are insufficient [13, 26]. In this context, breakthrough VSB is probably not a major cause of death in the present allo-HSCT setting, where beta-lactam non-susceptible VGSs and ARDS that associated with VSB are rare.

Some studies have recommended early or prophylactic vancomycin administration to prevent VSB [5, 12, 13]. However, considering our results, clinicians should be able to reduce that kind of AGPA administration in certain allo-HSCT settings, especially where beta-lactam non-susceptible VGS is not prevalent, such as Japan and Finland. Further　epidemiological investigations regarding VSB in various settings, including allo-HSCT settings are needed.

Our study had some limitations. First, this study was a single-center retrospective study. However, prospective, multicenter, or randomized trials to clarify appropriate antimicrobial usage for BSIs occurred in allo-HSCT settings, such as VSB, are becoming increasingly rare. Second, 182 of the 184 recipients (99 %) who received levofloxacin prophylaxis had FN, although levofloxacin prophylaxis has been reported to be effective in reducing FN incidence [28]. Fluoroquinolone prophylaxis is an accepted protocol for neutropenic patients with hematological malignancy and severe neutropenia [1, 28, 29], although the limitations of fluoroquinolone prophylaxis are also recognized [1, 30]. Therefore, analyzing the impact of levofloxacin breakthrough infections is important for current allo-HSCT settings. Third, susceptibility tests for APBL-VAs could not be performed against all 28 VGS strains. However, among the 22 stored VGS strains analyzed in this study, the susceptibility rates for APBL-VAs were high (Table 2). According to these data and the results of a previous report, high susceptibility rates for APBL-VAs against all the 28 strains are highly probable [11]. Forth, the risk factor of the VSB was not identified. That might be because the number of the recipients was not enough. Among, 92 recipients who were checked their oral conditions and had the data regarding them, the cumulative incidence of the VSB in the recipients with mucositis (21.3 %) tended to be higher than that in the recipients without it (9.7 %) (*P* = 0.16). Mucositis might be identified as the significant risk factor, if all the recipients were checked their oral conditions.

## Conclusions

In summary, the empiric addition of an AGPA, such as vancomycin to an APBL-VA-based regimen would not typically be needed for treating breakthrough VGS strains in this setting. The indication of adding an empiric AGPA to APBL-VA based regimen for treating VSB should depend on the local factors in each setting. In addition, breakthrough VSB is probably not a major cause of death in the allo-HSCT setting where the beta-lactam non-susceptible VGS strains and the ARDS that associated with VSB are rare.

## Abbreviations

allo-HSCT, allogeneic hematopoietic stem cell transplant; BSI, blood stream infection; VGS, viridans group streptococcus; FN, febrile neutropenia; IDSA, Infectious Diseases Society of America; APBL, anti-pseudomonal beta-lactam; APBL-VAs, APBLs with increased anti-VGS activity; AGPAs, anti-gram-positive agents; MIC, minimum inhibitory concentration; VSB, viridans streptococcal bacteraemia; ANC, absolute neutrophil count; MRSA, methicillin-resistant *Staphylococcus aureus*; VRE, vancomycin-resistant enterococci; AML, acute myeloid leukemia; MDS, myelodysplastic syndrome; PH-allo-HSCT, prior history of allogeneic hematopoietic stem cell transplantation; ARDS, acute respiratory distress syndrome; CLSI, Clinical Laboratory and Standards Institute; EC, evaluation cohort

## Additional files

Additional file 1: The result of risk factor analysis for levofloxacin breakthrough VSB among the 184 recipients. (DOCX 20 kb) Additional file 2: Clinical characteristics of each of the three groups in the evaluation cohort. (DOCX 47 kb)
