# Supplementary material for: Breakthrough viridans streptococcal bacteremia in allogeneic hematopoietic stem cell transplant recipients receiving levofloxacin prophylaxis in a Japanese hospital
Source: BMC Infect Dis. 2016 Aug 5;16:372. doi: 10.1186/s12879-016-1692-y (PMC4975918; doi:10.1186/s12879-016-1692-y)
Supplement: Additional file 1: — The result of risk factor analysis for levofloxacin breakthrough VSB among the 184 recipients. (DOCX 20 kb) [file 12879_2016_1692_MOESM1_ESM.docx]

Additional file 1 The result of risk factor analysis for levofloxacin breakthrough VSB among the 184 recipients

CBT, cord blood transplantation; RIC, reduced-intensity conditioning; MAC, Myeloablative conditioning; CI, confidence interval; PH-allo-HSCT, prior history of allogeneic hematopoietic stem cell transplantation.
