# Supplementary material for: Breakthrough viridans streptococcal bacteremia in allogeneic hematopoietic stem cell transplant recipients receiving levofloxacin prophylaxis in a Japanese hospital
Source: BMC Infect Dis. 2016 Aug 5;16:372. doi: 10.1186/s12879-016-1692-y (PMC4975918; doi:10.1186/s12879-016-1692-y)
Supplement: Additional file 2: — Clinical characteristics of each of the three groups in the evaluation cohort. (DOCX 47 kb) [file 12879_2016_1692_MOESM2_ESM.docx]

Additional file 2 Clinical characteristics of each of the three groups in the evaluation cohort

BSI, blood stream infection; VSB, viridans streptococcal bacteremia; AML, acute myeloid leukemia; MDS, myelodysplastic syndrome; CML, chronic myelogenous leukemia; ALL, acute lymphoblastic leukemia; HL, Hodgkin lymphoma; NHL, non Hodgkin lymphoma; ATLL, adult T cell leukemia/lymphoma; MPAL, mixed phenotype acute leukemia; SAA, severe aplastic anemia; ANCs, absolute neutrophil counts; PH-allo-HSCT, prior history of allogeneic hematopoietic stem cell transplantation; CBT, cord blood transplantation; rPBSCT, related peripheral blood cell transplantation; uBMT, unrelated bone marrow transplantation; RIC, reduced-intensity conditioning; MAC, myeloablative conditioning; HVG, host versus graft; GVHD, graft versus host disease; TAC, tacrolimus; MMF, mycophenolate mofetil; CsA, cyclosporine; sMTX, short-term methotrexate; VCM, vancomycin; CFPM, cefepime; PIPC/TAZ, piperacillin/tazobactam; MEPM, meropenem; AMK, amikacin.
